# Supporting recommendations for childhood preventive interventions for primary health care: elaboration of evidence synthesis and lessons learnt

**DOI:** 10.1186/s12887-021-02638-8

**Published:** 2021-09-08

**Authors:** Sophie Jullien, Gottfried Huss, Ralf Weigel

**Affiliations:** 1 Barcelona Institute for Global Health, University of Barcelona, Barcelona, Spain; 2 Free public child health Consultant, European Confederation of Primary Care Paediatricians, Lyon, France; 3 Friede Springer endowed professorship for Global Child Health, Witten Herdecke University, Witten, Germany

**Keywords:** Prevention, Screening, Child, Evidence-based medicine

## Abstract

**Background:**

Recommendations to prevent morbidity and mortality in children was a high priority for the editorial group of a WHO pocket book for primary health care in the European region. However, the benefit of preventive interventions is not always clear and recommendations differ across countries and institutions. Here, we summarize the existing recommendations and the most recent evidence on ten selected preventive interventions applied to children under five years to inform this group. In addition, we reflect on the process and challenges of developing these summaries.

**Methods:**

For each intervention, we systematically searched for current recommendations from the WHO, the United States Preventive Services Task Force, the workgroup PrevInfad from the Spanish Association of Primary Care Pediatrics, the Centers of Disease Control and Prevention, and the National Institute for Health and Care Excellence. Then, we systematically searched the sources above and the Cochrane library for relevant systematic reviews. For each topic, we reported the recommendations and the strength of the recommendation when and as reported by the authors. We summarized the main findings of systematic reviews with the certainty of the evidence as reported. Categorising the ten preventive interventions in three groups allowed narrative comparisons between similar types of interventions and between groups.

**Results and discussion:**

For the single interventions of providing vitamins D and K and topical fluoride there is overall a high degree of consensus between institutions for the evidence of their effectiveness. For the multiple interventions to prevent sudden infant death syndrome and unintentional injuries consensus was more variable as evidence of effectiveness is harder to ascertain. For the screening interventions the summaries of recommendations and evidence varied too. While institutions generally agreed in recommending for vision screening and against universal screening for language and speech delay and iron deficiency, they had some differences for pulse oximetry and autism.

The transparent and independent process shed light upon how institutions use existing evidence in their settings – common and different positions were accounted for and became visible. We also identified gaps and duplications of research. Our approach was a crucial starting point for developing the respective sections in the pocket book.

**Supplementary Information:**

The online version contains supplementary material available at 10.1186/s12887-021-02638-8.

## Background

Preventable morbidity and mortality among children constitute a critical global health matter. Preventive interventions are specific population- and individual-based interventions, which aim to protect, promote, and maintain health and well-being, and to minimize the burden of diseases, associated risk factors and disability [1]. Preventive care of children has a potentially huge benefit in decreasing childhood preventable morbidity and mortality. In the last decades, a broad list of preventive interventions targeting infants and children has been developed and implemented in the different health systems. While some of those interventions are evidence-based practice with proven benefit, others are common practice based on common sense or expert opinion, without clear evidence justifying effectiveness or benefit, resulting potentially in controversy. Each preventive intervention is however delivered at a cost for both the families and the health systems. There is a need to determine the safety and effectiveness of controversial preventive interventions, to optimize the protection of children and to optimize the use of limited health resources.

The World Health Organization (WHO) European Region is developing a new pocket book for primary health care for children and adolescents in Europe. To this end, the Integrated Management of Childhood Illness (IMCI) and recommendations around common childhood diseases are being revised, so that the pocket book provides updated recommendations applicable to the European setting, to ensure provision of quality services at primary health care level.

In this review, we aimed to summarize the existing recommendations and the most recent evidence on ten selected preventive interventions applied to children under 5 years of age to inform the WHO editorial group to make recommendations for health promotion in primary health care. The ten selected preventive interventions were: vitamin D prophylaxis in infancy, vitamin K prophylaxis in newborns, prophylaxis of caries with fluoride for children under 5 years, sudden infant death syndrome (SIDS) prevention, prevention of unintentional injuries in children under 5 years, vision screening in newborns and early childhood, newborn screening for critical congenital heart defects, screening of iron deficiency anaemia in early childhood, screening for autistic spectrum disorder (ASD) in early childhood, and screening for language and speech delay in children under 5 years.

In addition, we reflected on the process and challenges of developing recommendations based on available evidence and other determining factors, according to the type of recommendations.

## Methods

### Selection process of topics

The list of preventive interventions that we reviewed is neither an exclusive nor a comprehensive selection of preventive interventions for children under 5 years of age. We selected preventive interventions suitable for primary health care settings common in the European region, and applicable to primary health care physicians including paediatricians and other primary care providers. We followed a multi-step process with the aim to select most relevant topics for child health where evidence of benefit is controversial and for which evidence synthesis is most needed for decision making.

1. Pre-selection of preventive interventions: during the autumn meeting of the European Confederation of Primary Care Paediatricians (ECPCP) in October 2018 in Vilnius the 25 delegates representing ten countries completed a survey about the estimated magnitude of the benefit of 22 preventive interventions.
2. Exclusion of the interventions with high consensus on benefit: a steering group reviewed the survey results and identified six interventions with high consensus of benefit and decided to focus on those with controversial benefit.
3. The steering group presented survey findings to the WHO editorial core team of the pocket book to reach a final consensus and selected ten preventive interventions for evidence synthesis, based on priority research and feasibility criteria including time constraint.

### Formulation of key questions

For each topic, we formulated several key questions following the Participants, Intervention, Comparison, Outcomes (PICO) format whenever this was applicable. The main focus of the key questions addresses the effectiveness of the preventive interventions. We also assessed the balance between benefits and harms of the preventive interventions whenever this was applicable and feasible.

### Search methods

Firstly, we gathered existing recommendations for each preventive intervention, from sources we deemed relevant with potential applicability to the European context. We then intended to gather all available evidence to assess effectiveness and safety of the preventive interventions. We developed a research protocol that includes the search methods prior to starting the search.

#### Identification and selection of recommendations

For each topic, we systematically searched for current recommendations from the WHO, the United States Preventive Services Task Force (USPSTF), the workgroup PrevInfad from the Spanish Association of Primary Care Pediatrics (AEPap), the Centers for Disease Control and Prevention (CDC) in the US, and the UK’s National Institute for Health and Care Excellence (NICE) websites, by using key words (specified for each topic in the corresponding article) and by manual search [2–6]. We limited the search from the 1st January 2010 up to November 2019 and identified recommendations regardless of language. These websites as well as the references from the identified recommendations were checked for systematic reviews or relevant studies supporting their statements. In the case of USPSTF, we also checked the recommendations in progress for identification of any recommendations to be published soon.

We selected these sources in agreement with the expert group members in charge of developing the preventive chapter for the WHO pocket book for primary childcare. For topics with no or a limited number of recommendations by the above institutions and sources, we searched for additional sources that we cited in the corresponding article.

#### Identification and selection of supporting evidence

We systematically searched for systematic reviews commissioned or cited by the WHO, USPSTF, PrevInfad and NICE to inform their recommendations, published from 2010 up to date. For each topic, we also searched the Cochrane library for identification of relevant systematic reviews, by using key terms in titles, abstracts and keywords [7]. We screened titles and abstracts of the reviews retrieved from the search strategy for considering inclusion in this document, before looking at full-text reviews. We included all reviews addressing the topic as per the research question established. We also screened the Cochrane protocols retrieved by the search strategy, to identify systematic reviews to be potentially published in the near future. When a protocol was identified, we contacted the authors to enquire the status of the review and the intended date of publication of the systematic review. When no systematic review was found on a particular topic from the above sources, we considered including reviews published earlier than 2010 and conducted a literature search in Pubmed to identify any relevant review or trial. These additional resources were included as considered by the author of each topic so that a complete summary of the existing literature could be presented for each topic; they are cited in the corresponding articles.

### Data collection and data synthesis

#### Summary of recommendations

For each topic, we cited the recommendations identified from the different sources and the strength of the recommendation when and as reported by the authors. USPSTF classifies their recommendations in one out of five grades to describe the strength of the recommendation (Table 1) [8]. PrevInfad uses either the USPSTF method or the Grading of Recommendations Assessment, Development and Evaluation (GRADE) methodology and classifies their recommendations as strong or weak [9]. NICE considers the strength of the recommendation based on the GRADE approach; however, it is only reflected in the wording of the recommendation itself. Indeed, the words such as ‘offer’, ‘measure’, ‘advise’ or ‘refer’ are used in NICE documents to reflect a strong recommendation, while ‘consider’ is used to reflect a recommendation for which the trade-off between desirable and undesirable effects is less certain [10]. We specified in each article the methods used for grading the recommendations whenever it differed from the above methods.

**Table 1:** Grade recommendations from the USPSTF [8]

Grade	Definition	Suggestions for practice
**A**	The USPSTF recommends the service. There is high certainty that the net benefit is substantial.	Offer or provide this service.
**B**	The USPSTF recommends the service. There is high certainty that the net benefit is moderate or there is moderate certainty that the net benefit is moderate to substantial.	Offer or provide this service.
**C**	The USPSTF recommends against routinely providing the service. There may be considerations that support providing the service in an individual patient. There is at least moderate certainty that the net benefit is small.	Offer or provide this service only if other considerations support the offering or providing the service in an individual patient.
**D**	The USPSTF recommends against the service. There is moderate or high certainty that the service has no net benefit or that the harms outweigh the benefits.	Discourage the use of this service.
**I**	The USPSTF concludes that the current evidence is insufficient to assess the balance of benefits and harms of the service. Evidence is lacking, of poor quality, or conflicting, and the balance of benefits and harms cannot be determined.	Read the clinical considerations section of USPSTF Recommendation Statement. If the service is offered, patients should understand the uncertainty about the balance of benefits and harms.

#### Summary of the evidence

We summarized the main findings of systematic reviews in a narrative way and in summary tables. We also reported the certainty of the evidence as reported by the review authors. Cochrane authors used the GRADE approach for assessing the certainty of the evidence [11]. This consists of assessing several factors for each important and critically important outcome. Five factors can lower the certainty of the body of evidence: limitations in study design and execution, indirectness, imprecision, inconsistency and publication bias; and three factors can increase the certainty of the body of the evidence from observational studies: dose-response gradient, direction of plausible bias and magnitude of the effect. The certainty of the evidence is graded as high, moderate, low and very low for each assessed outcome. Authors of reviews commissioned by the USPSTF used another method developed by the USPSTF. It is based on the number, quality, size of studies, consistency of results among studies, and directness of the evidence, to grade the aggregate internal validity (quality) of the body of evidence for each key question as good, fair, or poor [8] (Table 2).

**Table 2:** Assessment of the quality of the evidence by USPSTF [8]

Grade	Definition
**Good**	Evidence includes consistent results from well-designed, well-conducted studies in representative populations that directly assess effects on health outcomes.
**Fair**	Evidence is sufficient to determine effects on health outcomes, but the strength of the evidence is limited by the number, quality, or consistency of the individual studies, generalizability to routine practice, or indirect nature of the evidence on health outcomes.
**Poor**	Evidence is insufficient to assess the effects on health outcomes because of limited number or power of studies, important flaws in their design or conduct, gaps in the chain of evidence, or lack of information on important health outcomes.

No statistical analysis was performed from the gathered evidence.

#### Groups of preventive interventions for narrative synthesis

We classified the ten preventive interventions into three groups, to allow narrative comparisons among similar types of interventions, and for reflection on the process and the challenges of developing recommendations based on available evidence:

- Single pharmacological interventions for preventing undesirable outcomes (primary prevention): vitamin D supplementation for preventing rickets and infections, vitamin K for preventing haemorrhage disease of the newborn, and fluoride for preventing dental caries.
- Multiple interventions for preventing an undesirable outcome (primary prevention): interventions addressing risk factors and promoting beneficial measures for preventing SIDS and unintentional injuries.
- Screening interventions to detect a condition early (secondary prevention): vision screening in newborns and early childhood, use of pulse oximetry for newborn screening of critical congenital heart defects, screening of iron deficiency anaemia, screening for ASD, and screening for language and speech delay.

## Results

We present the evidence synthesis for each of the ten preventive interventions in separate articles of this Supplement, including a comprehensive list of references of all the included sources. Table 3 summarizes the source and date of existing recommendations. For most topics, USPSTF and PrevInfad provided clear recommendations and supporting documents (systematic reviews) for informing their decisions. Although most of the topics were addressed by the CDC and NICE, accessing the documents supporting their recommendations was difficult, mostly due to a lack of references. We did not find WHO recommendations for three topics.

**Table 3:** Source and date of existing recommendations

Types of interventions	Interventions	WHO	USPSTF	PrevInfad	CDC	NICE	Others
**Single interventions**	**Vitamin D**	2019	No	2009^a^	2018	2008	Global consensus, 2016
	**Vitamin K**	2017	No	2010^a^	2018	2015	ESPGHAN, 2016 CADTH, 2015
	**Fluoride**	2018	2014^a^	2011^a^	2001	2014	AAP, 2014 NHS, 2017
**Multiple interventions**	**SIDS**	AAP^b^	AAP^b^	2016^a^	AAP^b^	2015	AAP, 2016^a^
	**Unintentional injuries**^**c**^	NA	NA	NA	NA	NA	NA
**Screening interventions**	**Vision screening**	No	2017^a^	2016^a^	2019	2015	AAP, 2016 RCPCH, 2019 UK NSC, 2013
	**Pulse oximetry for CCHD screening**	No	No	No	2018	2015	AAP, 2019 AEP, 2018 RCPCH, 2019 UK NSC, 2014
	**IDA screening**	2001	2015^a^	2011^a^	1998	No	AAP, 2010 UK NSC, 2017
	**ASD screening**	2019	2016^a^	2017^a^	2015	2013^d^	AAP, 2015 CTFPHC, 2019 UK NSC, 2012
	**Language and speech delay screening**	No	2015^a^	2017^e^	2019^d^	No	CTFPHC, 2016^e^ RCPCH, 2019

*Abbreviations*: *AAP* American Academy of Pediatrics, *AEP* Spanish Paediatric Association (Asociación Española de Pediatría), *ASD* Autistic spectrum disorder, *CADTH* Canadian Agency for Drugs and Technologies in Health, *CCHD* Critical congenital heart defects, *CDC* Centers of Disease Control and Prevention, *CTFPHC* Canadian Task Force on Preventive Healthcare, *ESPGHAN* European Society for Paediatric Gastroenterology Hepatology and Nutrition, *IDA* Iron deficiency anaemia, *NA* Not applicable, *NHS* National Health Service, *NICE* National Institute for Health and Care Excellence, *RCPCH* Royal College of Paediatrics and Child Health, *SIDS* Sudden infant death syndrome, *UK NSC* United Kingdom National Screening Committee, *USPSTF* United States Preventive Services Task Force, *WHO* World Health Organization

^a^These sources include both recommendations and supporting evidence through an independent systematic review

^b^These institutions support and refer to the AAP recommendations for this topic

^c^Due to the extent of this topic, a different approach was used for evidence synthesis. See corresponding article

^d^Some information in this topic, although no clear recommendations were found

^e^Documents on the broader area of developmental delay

We summarized the main characteristics of the supporting evidence (such as source of the evidence and main findings), the certainty of the evidence, the recommendations across the reviewed documents and the strength of the recommendations in Tables 4, 5 and 6.

**Table 4:** Summary of evidence and recommendations for single interventions

	Evidence					Recommendations	
Intervention	Type of interventions	Types of studies	Date	Findings	Certainty of the evidence^a^	Recommendations across reviewed documents	Strength of recommendations^a^
**Vitamin D**	Effectiveness of vit D supplementation for preventing nutritional rickets in term born children (clinical and radiological outcomes)	SR (4 trials, 1700 participants) RCT Surveillance data	SR 2007 Studies 1994 to 2004	Vit D supplementation prevents from developing rickets	High quality evidence according to European global consensus	- WHO: acknowledges effectiveness, but requests further research for specific recommendations - PrevInfad, CDC, NICE, Global European consensus recommend	- WHO: category 2 intervention - PrevInfad: Grade B - European global consensus: strong
	Effectiveness of vit D supplementation for improving bone mineral density (BMD)	SR (6 RCTs, 884 participants)	SR 2010 Studies 2004 to 2008	No effect on total body BMD or lumbar spinal BMD. Probably no effect on hip BMD and forearm BMD.	High certainty evidence Moderate certainty evidence		
**Vitamin K**	Effectiveness of vit K for preventing HDN (clinical outcome: bleeding)	SR (2 RCTs, only for classical HDN and IM)	Studies 1960’s	Effective	Not graded by Cochrane authors but referred as ‘poor methodological quality’ Graded as low-moderate by WHO	Recommended by all institutions	Strong (WHO, PrevInfad)
	Effectiveness of vit K for preventing HDN (biochemical outcomes)	SR (5 RCTs, only for classical HDN, IM and oral)	Studies 1990’s	Effective			
	Intramuscular versus oral	No RCT National surveillance data	Last 3 decades	Both intramuscular and oral effective	Not applicable		
**Fluoride**	Toothbrushing for preventing dental caries	SRs	SR 2019 Studies 1982 to 2014	Effective	Moderate	Recommended by all institutions	B recommendation (USPSTF); strength of the evidence I (NHS)

*Abbreviations*: *BMD* Bone mineral density, *CDC* Centers of Disease Control and Prevention, *HDN* Haemorrhage disease of the newborn, *NHS* National Health Service, *NICE* National Institute for Health and Care Excellence, *RCT* Randomized control trial, *SR* Systematic review, *USPSTF* United States Preventive Services Task Force, *vit* Vitamin

^a^As per the authors of the retrieved documents

**Table 5:** Summary of evidence and recommendations for multiple interventions to prevent SIDS and unintentional injuries

	Evidence					Recommendations	
Preventable outcomes	Type of interventions	Types of studies	Date	Findings	Certainty of the evidence^a^	Recommendations across reviewed documents	Strength of recommendations^a^
**SIDS**	Supine position Firm surface Overheating, head covering Room-sharing, bed-sharing Breastfeed on demand Pacifier nap and bedtime Etc.	No RCTs Case-control studies National pre and post intervention data	Most studies between 1990 and 2004	See corresponding article (10.1186/s12887-021-02536-z)	Overall low certainty of the evidence, but not for all risk factors	Overall, similar recommendations from the different institutions	A, B, C, I, not graded depending on the risk factors
**Unintentional injuries**	Against road traffic injuries: child restraint system, helmet, safety education of pedestrians, etc. Against drowning: pool fencing, life jackets, close supervision at all times, etc. Against poisoning: packaging, secure storage, poison control centres, etc. Against thermal injuries: smoke alarms, control of hot water, non-flammable fabrics, etc. Against falls: safety equipment, protective equipment, supervision, etc.	No RCTs Retrospective studies Surveillance data	Variable, see corresponding article (10.1186/s12887-021-02517-2)	See corresponding article (10.1186/s12887-021-02517-2)	Overall low certainty of the evidence, many recommendations based on good clinical practices.	Overall, similar recommendations from the different institutions	A, B, C, I, not graded depending on the risk factors

*Abbreviations*: *RCT* Randomized control trial, *SIDS* Sudden infant death syndrome

^a^As per the authors of the retrieved documents

**Table 6:** Summary of evidence and recommendations for screening interventions

	Evidence				Recommendations	
Screening interventions	Accuracy of screening tests (certainty of the evidence^**a**^)	Effectiveness of treatment (certainty of the evidence^**a**^)	Potential harms (certainty of the evidence^**a**^)	Benefits of screening: early detection for early intervention (certainty of the evidence^**a**^)	Recommendations across reviewed documents	Strength of recommen-dations^**a**^
**Vision screening**						
Newborn and small infants: ocular inspection and red reflex	Low sensitivity	High	Harmless examination	Essential	Recommended (PrevInfad, CDC, NICE, RCPCH)	Not graded
Children 6–59 months: screening amblyopia and its risk factors	Accurate tools for detecting amblyopia and risk factors (low certainty of the evidence)	Overall effective	Some psychological harms related with patching (uncertainty of the evidence)	Indirect evidence of moderate net benefit (moderate certainty of evidence)	- In < 3 years: insufficient evidence (USPSTF, PrevInfad, CDC) - In 3 to 5 years: screening recommended (USPSTF, PrevInfad, CDC, AAP, RCPCH, UK NSC)	I statement B recommen-dation
**Pulse oximetry for detecting CCHD**						
Use of pulse oximetry for detecting CCHD	SR (16 prospective and 5 retrospective studies): for a threshold of < 95% for positivity: sensitivity 76.3% (low certainty evidence); specificity 99.9% (high certainty evidence)	Effective	Safe and harmless tool Unnecessary investigations (not invasive), potentially admissions and familiar anxiety for false positive cases	Overall beneficial	- Screening recommended by AEP (Level A evidence), CDC, AAP - Insufficient evidence to recommend screening according to RCPCH, UK NSC	Not available
Timing of pulse oximetry for detecting CCHD	- Early < 24 h (SR, 8 studies): sensitivity 79.5% and specificity 99.6%. - Late > 24 h (SR, 11 studies): sensitivity 73.6% and specificity 99.9%. - False positive rate significantly lower among newborns screened > 24 h (0.06% versus 0.42%; *P* = 0.027)			Early diagnosis leads to timeliness of treatment. Early screening leads to higher false positive rates. But most false positive cases are indicative of other severe disorders that may also require prompt diagnosis and treatment.	- Early screening (< 24 h) recommended by AEP (Level B evidence) - Late screening (> 24 h) recommended by CDC, AAP	Not available
**Universal screening for detecting IDA**						
IDA screening	No suitable test for IDA screening that is non-invasive with high accuracy for detecting IDA	Lack of evidence on the effects of treatment of IDA for improving growth, cognitive and psychomotor development outcomes	Lack of evidence that evaluate harms of overall screening. Adverse effects associated with oral iron: no differences found between children receiving iron and placebo.	Lack of evidence	- Recommendation against screening by PrevInfad, the UK NSC, USPSTF - Screening recommended by AAP	Strong recommen-dation (PrevInfad); I statement (USPSTF)
**Universal screening for detecting ASD**						
In young children for whom no concerns of ASD have been raised	Accurate tools	Some evidence of benefit of early interventions applied to children with ASD identified due to developmental concerns. No evidence on the effectiveness of interventions applied to children with ASD detected through screening.	Limited evidence assessing harms of screening or interventions for ASD. Potential harms are likely to be small. However, interventions can be associated with important burden for the families in terms of time and resources.	Overall lack of evidence	- Screening recommended by CDC, AAP - Recommendation against universal screening by PrevInfad, UK NSC, Canadian Task Force on Preventive Healthcare - Insufficient evidence to assess the balance of benefits and harms of screening for ASD by USPSTF	Weak recommen-dation (PrevInfad); I statement (USPSTF)
**Universal screening for detecting language and speech delay**						
In young children for whom no concerns about their speech or language have been raised	Wide variation in accuracy. No single screening tool with best characteristics for screening.	Evidence that targeted interventions improve some measures of speech and language delay and disorders. However, there is no evidence on the effectiveness of such interventions in children detected by screening with no specific concerns about their speech or language prior to screening	Burden for the families in terms of time, resources, anxiety. No evidence on the harms of screening for language and speech delay in primary care settings Limited evidence assessing potential harms of interventions.	Lack of evidence	- Recommendation against universal screening by, UK NSC, Canadian Task Force on Preventive Healthcare - Insufficient evidence to assess the balance of benefits and harms of screening for ASD by USPSTF	Strong recommen-dation (Canadian Task Force); I statement (USPSTF)

*Abbreviations*: *AAP* American Academy of Pediatrics, *AEP* Spanish Paediatric Association (Asociación Española de Pediatría), *ASD* Autistic spectrum disorder, *CCHD* Critical congenital heart defects, *CDC* Centers of Disease Control and Prevention, *IDA* Iron deficiency anaemia, *NICE* National Institute for Health and Care Excellence, *RCPCH* Royal College of Paediatrics and Child Health, *SR* Systematic review, *UK NSC* United Kingdom National Screening Committee, *USPSTF* United States Preventive Services Task Force

^**a**^As per the authors of the retrieved documents

### Single interventions for preventing an undesirable outcome

Three articles of this Supplement reviewed single interventions. We found that all the international institutions we looked at (including the WHO, PrevInfad, NICE and European consensus) recommend supplementation with vitamin D for preventing rickets, administration of vitamin K for preventing the haemorrhage disease of the newborn, and use of topical fluoride for preventing caries. The strength of the recommendation for the main statement is strong or grade B for each intervention and is based on moderate to high certainty of the evidence for vitamin D supplementation and use of fluoride. Between the three interventions (vitamin D, vitamin K and topical fluoride), there are substantial differences in the time when trials or systematic reviews that support the evidence were conducted. For example, studies assessing the effectiveness of vitamin K for preventing the haemorrhage disease of the newborn were conducted in the 1960s and 1990s, while assessment of the use of topical fluoride for preventing caries come from more recent trials (up to 2014) with a systematic review conducted in 2019.

Although all institutions recommend these interventions in general, we noted a lack of data and an overall lower certainty of the evidence to inform specific aspects for each of these three interventions including doses and duration of supplementation. For example, there is a lack of evidence to inform whether vitamin D supplementation should be given only during the first 12 months of life or beyond, to inform what is the optimal regimen of oral vitamin K (doses and number of doses), or to inform which fluoride concentration is optimal in toothpastes for preventing caries and for limiting adverse effects.

### Multiple interventions for preventing an undesirable outcome

Two articles of this Supplement assessed the impact of several interventions for preventing either SIDS or unintentional injuries. In both cases, there were no randomised controlled trials (RCTs) and the evidence came from retrospective analyses (mainly case-control studies) and surveillance data only. Certainty of the evidence varied from low (mainly) to high, and the strength of the recommendation varied from strong to weak or insufficient (A, B, C, D or I) for the risk factors assessed. Overall, the different institutions recommend similar preventive interventions based on the available evidence and good practice recommendations based on common sense.

### Screening interventions for early detection of a disorder

Five articles of this Supplement summarize the evidence and existing recommendations for screening interventions.

For vision screening, we found that all the institutions we looked at recommend screening all newborns for congenital cataract and retinoblastoma through ocular inspection and red reflex examination. Although the screening has low sensitivity, it is a harmless examination that is widely accepted due to the severity of both diseases, and both diseases clearly benefit from early detection and treatment. All institutions also recommend vision screening among children between three and 5 years of age, based on indirect evidence of moderate net benefit.

All the institutions we looked at recommend against universal screening for detecting iron deficiency anaemia and against universal screening for detecting language and speech delay (or make the statement of insufficient evidence to assess the balance of benefits and harms of screening for language and speech delay). The strength of the recommendation for both screening interventions was judged as strong, and was based on the lack of a suitable screening tool and the lack of evidence of benefits from early interventions applied to children detected by screening. The American Academy of Pediatrics is the exception, and recommends universal screening for detecting iron deficiency anaemia despite the lack of evidence.

For the remaining two screening interventions, institutions have discrepant views. Some institutions do not recommend pulse oximetry in all newborns because its less than optimal sensitivity leads to false positive results that require unnecessary referral of cases representing a burden to families and the health system. Institutions recommending it however argue that most of the patients with false positive tests may have other severe clinical conditions and would benefit from prompt recognition and treatment, at least within the first 24 h of life. Views of institutions on universal screening for detecting ASD are conflicting too. Although screening tools do identify children aged 12 to 36 months with ASD accurately, and small studies show a benefit of early treatment, no evidence on the effectiveness of interventions applied to children with ASD detected through screening was found.

## Discussion

Overall, we found that there is high consensus among institutions for recommending single preventive interventions, for which there is moderate to high evidence that such interventions are effective. However, for these single interventions, the lack of data or the low certainty of the evidence regarding doses, frequency or duration of supplementation results into less clear recommendations and ultimately lesser consensus between institutions. Indeed, while no institution is arguing that vitamin K is effective for preventing haemorrhagic disease of the newborn, national policies are recommending different regimens with regard to route, dose and frequency. This is likely to be explained by the limited data from studies that would directly compare different regimens. In this case, we acknowledge that prospective and comparative trials are challenging to be conducted in the future, due to the low incidence of the outcome and above all due to the severity of the outcome.

For SIDS and unintentional injuries, assessing the effectiveness of preventive interventions is difficult due to the nature of the outcome (death or severe morbidity) and the multiple causes involved. For obvious ethical reasons, no RCT can be performed to assess the impact of selected risk factors on the outcome. Evidence is therefore mainly based on retrospective case control studies and surveillance data. This leads to difficulties in differentiating associations from causality between risk factors and outcomes. Furthermore, the multi-causality of the outcome (several risk factors are associated with the outcome) adds difficulties in establishing the contribution of each risk factor. In addition to the association between risk factors and the outcome (SIDS or unintentional injuries), there is overall limited evidence assessing the effect of the recommendations through knowledge, attitude and practices.

The assessment of screening tools seemed to be more controversial than for the single or multiple interventions, with a higher degree of disagreement between institutions. This is likely to be explained by the fact that more aspects must be considered when evaluating the balance between benefits and harms of universal screening than of single interventions. For interventions, the main factors to assess and balance are the effectiveness of the intervention to prevent the undesirable outcome and potential adverse effects associated with the intervention. In contrast, for screening, there is a need to assess the effectiveness of the screening tool to detect the condition, the potential harms derived from the screening tool and treatment, and the treatment effectiveness. In addition, outcomes following early detection and treatment versus the time of presenting signs or symptoms must be considered. Furthermore, the significance and consequences of false positive and false negative cases vary between settings with different prevalence of the disease and with different health care system. As the complete set of criteria for a good screening programme that was adopted by the WHO needs to be assessed, economic and other health system related factors need consideration too [12].

Apart from the immediate findings, the approach we used for this evidence synthesis is valuable in several other ways.

First, providing both aspects (summaries of existing recommendations and evidence) transparently and independently informs clinicians, researchers and policymakers about important research gaps. Where are formal systematic reviews required and where is field research needed? When we reviewed preventive interventions, it became clear that field research through new trials is needed most. Examples are trials to elucidate the optimal duration of vitamin D supplementation for preventing rickets, studies to determine the benefits of early detection and treatment of children with ASD compared with those identified with developmental concern, or research to find a suitable screening tool for iron deficiency anaemia.

Second, the review proved to be a unique opportunity to summarize what is already known across ten controversial and highly important preventive interventions for child health in a single document. It is a crucial starting point when a group or institution intends to develop or update recommendations. Indeed, this approach not only allows the identification of research gaps, but also areas for which recent systematic reviews have already been conducted and areas for which no further evidence might be required. In addition, we noticed that sometimes several institutions committed their own systematic reviews in order to inform the development or update of a recommendation, leading to several groups conducting independent systematic reviews in parallel and addressing the same research question. This represents a potentially avoidable duplication of work, wasting resources.

Third, our approach may facilitate ownership of the prevention chapters in the pocket book by institutions such as paediatric societies. We systematically looked at existing recommendations from credible European institutions, national and international, but also non-European institutions, for generalizable and applicable conclusions. By taking into consideration their views and positions and by making our methods clear, the content of the final product may become more acceptable to the target audience. Moreover, presenting an overview of the existing recommendations also sheds light on how various European institutions use and apply existing evidence to their settings – common and different approaches and positions become visible.

Finally, we complemented the European-focused summary of existing recommendations with an independent body of evidence from relevant systematic reviews of the Cochrane library. Separating the evidence from the recommendations allows the editorial group to gauge how different institutions considered factors such as acceptability, feasibility, potential harms, cost and others.

This review has limitations. There was a limited timeline that was defined by the editorial group and editorial process of the WHO pocket book. To address this, a single author was identified for conducting the literature search, data extraction and data synthesis. When we found no systematic review addressing one of our key questions, or when a systematic review was found but was outdated, it was not possible to conduct a needed systematic review ourselves. In addition, it was not possible to assess the methodological quality of included systematic reviews and guidelines using the AMSTAR-2 tool and the AGREE tool respectively.

The strengths of this review are the alignment to the principles of good evidence synthesis for policy – inclusive, rigorous, transparent and accessible [13, 14]. This review is inclusive, comprising of a range of relevant sources for both available evidence and recommendations. We believe that findings are relevant and useful to policymakers, especially for guiding those involved in the health promotion in primary health care for the paediatric pocket book for the WHO European Region. Although these evidence summaries do not assess external validity, we focused on gathering relevant sources from the European or similarly applicable settings when it comes to recommendations. The editorial group is invited to take this into consideration when moving from evidence to recommendations. We used a systematic and rigorous approach for all the topics. The study was conducted by an independent author and peer-reviewed for ensuring good quality. We developed a protocol prior to the evidence synthesis, reported the methods we followed, and acknowledged the limitations of our work with transparency. Finally, timeliness is key, and despite the time constraint, this compilation of evidence synthesis was made available in due time for its use by the WHO paediatric pocket book panel.

## Conclusions

The practice of well childcare in European countries consists of promotion of healthy nutrition and lifestyles and different methods in primary and secondary prevention of diseases. The rationale for this supplement is a response to a concrete need: to provide a WHO editorial group with a summary of evidence about selected preventive interventions with controversial benefit from a wide range of resources. This paper describes the methods used in each of the following articles, summarizes and analyses the existing recommendations and the underlying evidence for single and multiple preventive interventions and screening. In addition, it identifies lessons learnt from the process that might be useful for similar endeavours.

## Supplementary Information

**Additional file 1.** List of abbreviations.

## Data Availability

Not applicable.
