# Supplementary material for: Supporting recommendations for childhood preventive interventions for primary health care: elaboration of evidence synthesis and lessons learnt
Source: BMC Pediatr. 2021 Sep 8;21(Suppl 1):356. doi: 10.1186/s12887-021-02638-8 (PMC8424794; doi:10.1186/s12887-021-02638-8)
Supplement: Supplementary file 1 — Additional file 1. List of abbreviations. [file 12887_2021_2638_MOESM1_ESM.pdf]

List of abbreviations

AAO	American Academy of Ophthalmology
AAP	American Academy of Pediatrics
ABA	Applied behaviour analysis
ACCF	American College of Cardiology Foundation
AEPap	Spanish Association of Primary Care Pediatrics
AHA	American Heart Association
AOR	Adjusted odds ratio
ASD	Autistic spectrum disorder
CADTH	Canadian Agency for Drugs and Technologies in Health
CCHD	Critical congenital heart defect
CDC	Centers of Disease Control and Prevention
CHD	Congenital heart defect
CI	Confidence interval
CRP	C-reactive protein
DALYs	Disability-adjusted life years
dmfs	Decayed, missing and filled surfaces in primary dentition
dmft	Decayed, missing and filled teeth in primary dentition
ECPCP	European Confederation of Primary Care Paediatricians
EIBI	Early intensive behavioural intervention
eLENA	e-Library of Evidence for Nutrition Actions
EPA/UNEPSA	European Paediatric Association, the Union of National European Paediatric Societies and Associations

ESAT	Early Screening of Autistic Traits Questionnaire
ESPGHAN	European Society for Pediatric Gastroenterology Hepatology and Nutrition
FYI	First Year Inventory
GRADE	Grading of Recommendations Assessment, Development and Evaluation
HDN	Haemorrhagic disease of the newborn
ID	Iron deficiency
IDA	Iron deficiency anaemia
IM	Intramuscular
IMCI	Integrated Management of Childhood Illness
INR	International normalised ratio
IQ	Intelligence quotient
IU	International units
LR	Likelihood ratio
M-CHAT	Modified Checklist for Autism in Toddlers
M-CHAT-R	Modified Checklist for Autism in Toddlers-Revised
M-CHAT-F	Modified Checklist for Autism in Toddlers with Follow-Up
M-CHAT-R/F	Modified Checklist for Autism in Toddlers–Revised with Follow-Up
MD	Mean difference
NICE	National Institute for Health and Care Excellence
NPV	Negative predictive value
OR	Odds ratio
PIVKA-II	Prothrombin induced by vitamin K absence-II
PO	Pulse oximetry

ppm	Parts per million
PPV	Positive predictive value
RCPCH	Royal College of Paediatrics and Child Health
RCT	Randomized controlled trial
RD	Risk difference
RR	Relative risk
RUSP	Recommended uniform screening panel
SACHDNC	Secretary's Advisory Committee on Heritable Disorders in Newborns and Children
SACS	Social Attention and Communication Study
SIDS	Sudden infant death syndrome
SMD	Standardized mean difference
SUID	Sudden unexpected infant death
UK NSC	UK National Screening Committee
USPSTF	US Preventive Services Task Force
VABS	Vineland Adaptive Behaviour Scale
VIP study	Vision In Preschoolers study
VKDB	Vitamin K deficiency bleeding
WHO	World Health Organization
YACHT	Young Autism and Other Developmental Disorders Check-up Tool
